# Telehealth Training and Education for Allied Health Professionals: A Scoping Review

**DOI:** 10.1089/tmr.2024.0083

**Published:** 2025-03-19

**Authors:** Krithika Anil, Adam Bird, Kate Bridgman, Shane Erickson, Jenny Freeman, Carol McKinstry, Christie Robinson, Sally Abey

**Affiliations:** ^1^ School of Health Professions, University of Plymouth, United Kingdom.; ^2^ School of Allied Health, Human Services and Sport, La Trobe University, Victoria, Australia.; ^3^ School of Health and Biomedical Sciences, RMIT University, Victoria, Australia.

**Keywords:** telehealth, allied health professionals, education, training

## Abstract

**Background:**

With the growing adoption of telehealth in allied health disciplines, establishing clear training and education standards is crucial. This review aims to map the current training and education that has been delivered to support development of telehealth competencies in allied health professionals. This scoping review extends our previous review with an updated search.

**Methods:**

The Population Concept Context framework was used, and the following databases were searched: MEDLINE, CINAHL, PsychInfo, Cochrane, EMBASE, Web of Science, PEDro, United Kingdom Health Forum, WHO, Health Education England, and all U.K. and Australian Allied Health Profession (AHP) professional bodies.

**Results:**

Out of 1,05,980 articles, 12 met the inclusion criteria. Training and education differed greatly, with no definite pattern in teaching approaches. Three articles used standardized questionnaires for training and education assessment, while the remaining articles used author-designed assessments. Eight articles reported that participants achieved the targeted telehealth competencies, and five reported improved attitudes toward telehealth following training and education. Articles recommended various factors that may improve telehealth training and education outcomes, which included a combination of online and face-to-face methods, interprofessional training, consolidation of their skills through supervised clinical work, and separating video and telehealth competencies.

**Discussion:**

This scoping review represents the first comprehensive exploration of telehealth training and education across allied health disciplines. While articles yielded generally positive outcomes, the absence of standardized methods prompts questions about its efficacy. Research should focus on developing evidence-based curriculums informed by pedagogic practices tailored to allied health needs.

## Introduction

### Background

The term telehealth refers to the delivery of health care services using information and communication technologies for assessment, management, and prevention of health-related conditions.^1^ Relatively rapid improvements in technology during the Information Age and Fourth Industrial Revolution have enabled increasingly sophisticated interactions between service providers and users. However, there is significant variability in utilization of telehealth within, and between, the respective Allied Health Professions (AHPs).^2^ The utilization of telehealth as a delivery mode, alongside and integrated with in-person services, will continue to evolve over time, particularly as technologies mature and the broad benefits of telehealth including convenience and increased service availability are recognized by both service users and AHPs.

The continued implementation and development of telehealth services requires support from all stakeholders connected to health service delivery. At a health service organization level, these include ensuring the AHP workforce possesses the required skills, knowledge, and behaviors to provide safe, efficient, and effective telehealth services, underpinning by a clear strategy and determination of roles and responsibilities across the organization.^3^

During the COVID-19 pandemic, many clinicians had limited or no prior experience using telehealth^4,5^ and hence had not considered or acquired the required competencies, leading to feelings of uncertainty, fear, and apprehension.^6^ To deliver safe and effective telehealth services, AHPs require additional skills and behaviors to those required for in person consultations. There is an additional need to educate AHP students in the use of telehealth.^7^ Bridgman et al.^8^ synthesized the literature related to the perspectives of allied health students on clinical placements that incorporated telehealth. Although little has been published on this topic, a key finding was that considerable preparation is needed for students to use telehealth safely and professionally.

Our recent scoping review^9^ mapped telehealth competencies published by allied health disciplines and derived a set of competency themes currently in use. Eight overarching themes emerged that were related to the delivery of telehealth consultations, namely clinical reasoning, communication, effectively using technology, person-centered care, practice-based assessments and intervention knowledge, skills and behaviors, privacy, security, and safety, professionalism, and setting up the technology. There were three additional competency themes that outlined considerations related to health service management: digital infrastructure, informing practice, and management.

### Objectives

The current article aims to extend our recent review^9^ by addressing the question “*What training or education (inclusive of practice placements) has been delivered to support development of telehealth competencies in AHPs?*” We believe that this review is the natural progression of our previous work^9^ and will provide a foundation for AHP regulators, peak bodies, and training institutions to consider how to define or develop individual discipline (and potential cross-disciplinary) competency standards related to telehealth.

## Methods

A summary of the scoping review method is described below, with the detailed method of this scoping review reported in an earlier published article.^9^

### Ethics statement

It is a literature-based study; therefore, neither approval by the institutional review board nor the obtainment of informed consent is required.

### Study design and eligibility criteria

A scoping review was undertaken using the Population Concept Context framework^10^ and registered with Open Science Framework (https://osf.io/vrp62). This review included articles from 2012 to August 2023 that focused on at least one AHP, as specified by U.K.-based^11,12^ and Australian-based sources^13,14^ in Table 1. Competencies were broadly defined as the knowledge, skills, and behaviors needed to deliver AHP services efficiently and professionally.^15^ Telehealth technology must have been used synchronously, and all study designs and gray literature were included. Theses and books were excluded. Articles must have included AHP telehealth training and/or education.

**Table 1.** Allied Health Professions Included in This Scoping Review

Arts therapy	Operating department practitioners
Audiology	Optometry
Biomedical scientists	Orthoptists
Chiropractic	Orthotics/prosthetics
Chinese medicine practitioners	Osteopathy
Clinical scientists	Paramedic practitioners
Credentialed diabetes educators	Pedorthist
Dietetics	Perfusion
Diversional therapists	Pharmacy
Drama therapists	Physiotherapy
Exercise physiology	Podiatry
Genetic counseling	Psychology
Hearing aid dispensers	Rehabilitation counseling
Medical radiations/radiographers	Social work
Music therapy	Sonography
Occupational therapy	Speech pathology

### Information sources and search strategy

The following databases were searched: MEDLINE, CINAHL, PsychInfo, Cochrane, EMBASE, Web of Science, and PEDro. The websites of United Kingdom Health Forum, World Health Organisation (WHO), Health Education England (now called National Health Service England (NHSE)), and all United Kingdom (U.K.) and Australian AHP associations were also searched. Please see the Supplementary Data for search strategy and terms.

### Selection process and data synthesis

**FIG. 1.**
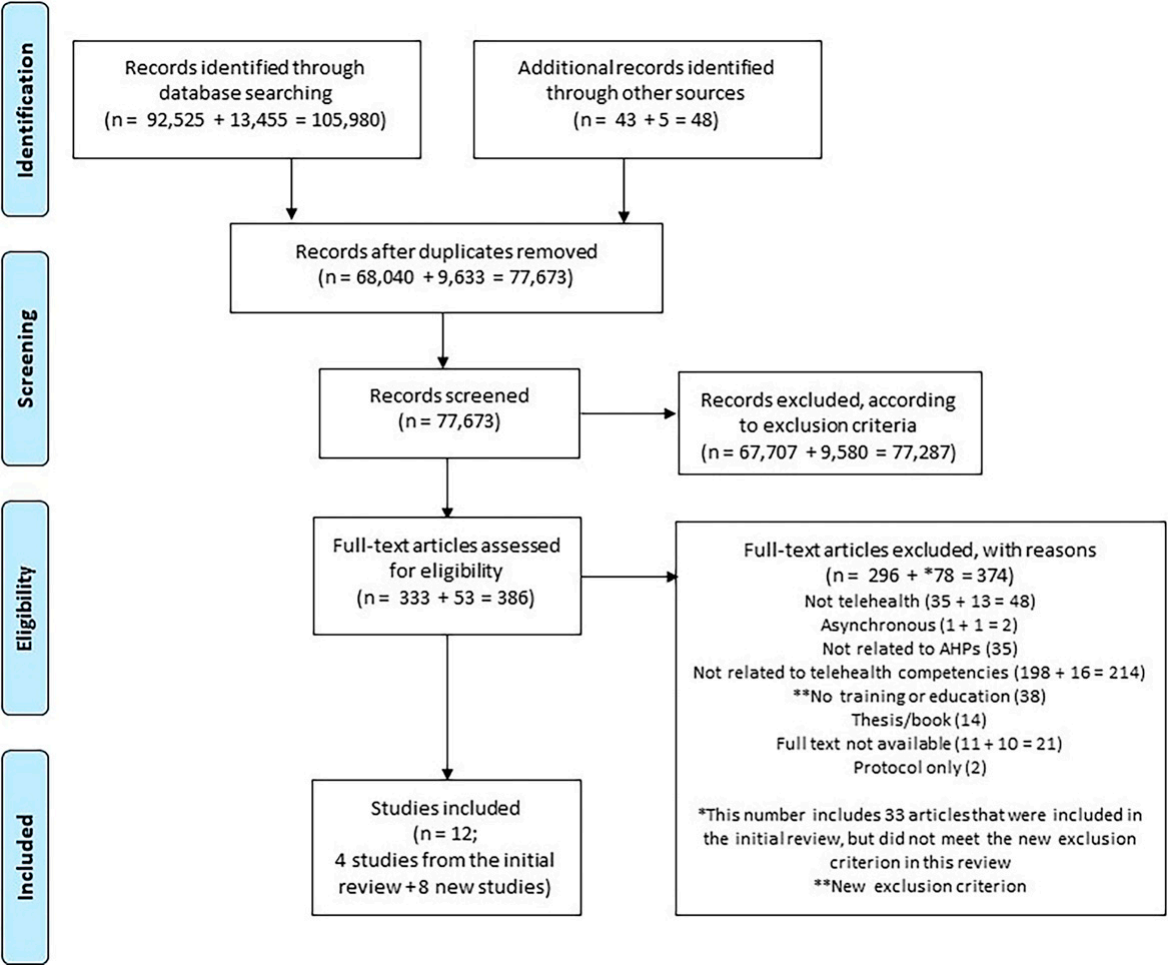
PRISMA flowchart of included articles; the initial search produced 1,05,980 articles that were carefully screened and resulted in 12 final articles.

Following the search, all identified references were reviewed using the online Rayyan tool.^16^ After removal of any duplications, titles and abstracts were divided and screened independently by the coauthors against the inclusion criteria. The research team includes experts from multiple AHP areas: physiotherapy, podiatry, speech pathology, and occupational therapy. This ensured article selection and analyses were not based on the perspective of a single profession. An agreement check was conducted after the abstract screening, where each team member was paired and checked 10% of each other’s screening. To ensure that cultural understanding was aligned, a U.K. team member was paired up with a team member from Australia. The full texts of potentially eligible studies were assessed independently by each coauthor against the inclusion criteria. Any disagreements between team members were resolved through discussion. Figure 1 displays our PRISMA flowchart.

Narrative data extraction was conducted using an Excel spreadsheet to extract data about telehealth training and education related to training/education description and schedule, context, use of any assessments/measurements, technology used, and related competencies. Any disagreements between team members were resolved through discussion. The findings are presented narratively in the results section aided by appropriate tables and figures.

The Hawker et al.^17^ critical appraisal tool was used to assess the included articles, which is useful to compare studies with different designs, as is the case with the current review. The tool assigns a total score based on the quality of the following: abstract and title, introduction and aims, method and data, sampling, data analysis, ethics and bias, generalizability, and implications. The scores are then divided into low (9–17), fair (18–26), and high (27–36).

## Results

Twelve articles were included in this scoping review (Fig. 1, see Table 2 for article characteristics, including quality scores). This number includes articles identified by the previous review^9^ (*n* = 4) and articles identified by the updated search (*n* = 8). Ten articles were research studies, which included cohort studies^19,23,25,27,28^ (*n* = 5), quasi-experimental studies^18,22^ (*n* = 2), case studies^24,29^ (*n* = 2), and a cross-sectional study.^26^ The remaining two articles were reports that described telehealth training and education without any evaluation.^20,21^ The majority of the articles originated from the United States^18,19,21,23,25–29^ (*n* = 9), and the remaining articles originated from Israel,^22^ the United Kingdom,^20^ and Vietnam.^24^ The 10 research articles were assessed for their quality, where seven articles were deemed “Fair”^18,19,22,24–27^ and three were “High.”^23,28,29^ The studies classified as “Fair” had insufficient consideration to potential biases in their study design and unclear generalizability of their training and education procedures. This was primarily due to the minimal reporting on the rationale for learning outcomes selection. The studies rated as “High” had robust methods, coherent reporting of findings, and clear implications. Overall, these quality scores highlight the need for more articulated methods and clear justification for telehealth training and educational outcomes in ensuring the reliability and applicability of the results.

**Table 2.** Article Characteristics

Authors (date)	Country of origin	Aim	Study design	Profession	Quality (score)
Baalmann et al. (2023)^18^	United States	To examine whether an interprofessional simulation using telehealth technology would provide medical and pharmacy students the opportunity to practice, develop, and grow in their confidence and skills of working collaboratively and disclosing medication errors.	Quasi-experimental	Pharmacy, Medicine	Fair (25)
Bautista et al. (2020)^19^	United States	To describe the development, implementation, and evaluation of an interprofessional rotation for third year pharmacy and medical students focused on telehealth outreach to patients at high risk for delaying care.	Cohort	Pharmacy, Medicine	Fair (20)
Bishop et al. (2013)^20^	United Kingdom	To describe the PhysioDirect system used in the trial and how physiotherapists were trained and supported to use the system and deliver the PhysioDirect service	Report (Not a study)	Physiotherapy	N/A (Not a study)
Crockett et al. (2020)^21^	United States	To describe the impact, changes, and outcomes achieved by a large, multifaceted, applied behavioral analysis clinical program that has (a) ongoing data that forecasted and tracked changes, (b) staff well practiced with data-based shifts in operations (behavior), and (c) up-to-date information (data) on policy and regulations.	Report (Not a study)	Psychology	N/A (Not a study)
Gafni-Lachter et al. (2023)^22^	Israel	To examine the effectiveness of an interprofessional telehealth course based on a collaborative needs assessment of professionals working in community-based child-development units.	Quasi-experimental	Psychology, Social Work, Speech Pathology, Physiotherapy, and Occupational Therapy	Fair (25)
Gifford et al. (2012)^23^	United States	To describe one model for delivering behavioral telehealth training of competencies and evaluate its effectiveness on developing those competencies.	Cohort	Counseling and Psychology	High (29)
McAllister et al. (2022)^24^	Vietnam	To explore the viability of online learning to continue clinical education (CE) of speech pathology students in Vietnam during the pandemic.	Single case/case series	Speech Pathology	Fair (20)
Pittman et al. (2023)^25^	United States	To describe a pilot telehealth interprofessional model used to educate students and provide patient care, outlining preliminary data about its effectiveness, and provide recommendations for future research and practice.	Cohort	Pharmacy, Social Work, and Dietetics	Fair (18)
Rohrig et al. (2022)^26^	United States	To compare online interprofessional practice to face-to-face practice, in nursing/allied health students.	Cross-sectional	Physiotherapy, Nursing, Occupational Therapy, Dietetics, Pharmacy, Speech Pathology	Fair (24)
Rude et al. (2023)^27^	United States	To develop and implement a simulation allowing students to practice interprofessional communication and assess the simulation’s impact on students’ confidence in providing pharmacy-related interventions to another health care professional via telephone.	Cohort	Pharmacy	Fair (26)
Tokumaru et al. (2023)^28^	United States	To evaluate how pharmacy and nursing students can practice telehealth as an interprofessional team while located in different areas using simulation	Cohort	Pharmacy and Nursing	High (27)
Vyas et al. (2022)^29^	United States	To assess the impact of role modeling, telehealth-based simulations, and formative feedback on student social and emotional development.	Single case/case series	Pharmacy	High (29)

### Competency themes

Tables 3 and 4 outline the competency themes described in our previous article.^9^ The framework showcases a consolidation of telehealth knowledge, skills, and behaviors from the articles within our scoping review rather than formally agreed competencies. Therefore, they are referred to as “competency themes” rather than “competencies.” Table 3 presents competency themes regarding the delivery of telehealth consultations, while Table 4 presents competency themes regarding telehealth service management. Two new competency themes were identified within this review and are underlined within Table 3. They relate to adapting resources for telehealth and developing a therapeutic alliance with service users.

**Table 3.** Competency Themes Relating to the Delivery of Telehealth Consultations

Delivery of telehealth consultations
Clinical reasoning
Check whether a telehealth consultation is an appropriate medium based upon the service-user’s health condition^30–32^
Know how to assess during a telehealth consultation whether in-person is required instead^20,30,33–37^
Know how to support service-users who have not previously used remote technologies^38,39^
Know whether telehealth is an appropriate medium to communicate updates to service-users^22,40^
Know how to select or adapt suitable resources for telehealth^22^
Communication
General communication
Know how to develop a contingency plan in case of technical failures and communicate this with the service-user^23,33^
Know how to work with the service-user to develop a communication plan during, and outside of, telehealth consultations as needed^41^
Preconsultation communication
Ensure there is a plan to resolve the issue of becoming disconnected with the service-user^21^
Explain the implications and limitations of telehealth versus in-person to the service-user when offering a telehealth service^31,42^
Know how to navigate failed communications, such as miscommunication, equipment failure, or service-user’s loss of faith in the telehealth service^42^
Make the service-user aware of their rights and responsibilities when receiving a telehealth consultation, including their right to refuse telehealth^43^
Prepare the client for the telehealth consultation by providing relevant and explicit information about the timing, technology, and instructions^33^
During consultation communication
Be proactive in ensuring the client understands the telehealth services being offered and understanding their service preferences^41,44–46^
Know how to address nonverbal cues when using telehealth^23,21,35,38,41,47,48^
Know how to effectively communicate via telephone as needed for a telehealth consultation^27,22,20,49^
Know how to effectively teach the service-user the basics of technology used for the telehealth consultation^38,50^
Know how to explicitly introduce yourself and check the client’s identity^33,40^
Know how to proactively and thoughtfully engage with the client using telehealth^29,20,34,37,39–41,44,49–51^
Post-consultation communication
Document any changes to assessments that were adapted for telehealth^36,45^
Effectively using technology
Ensure you are familiar with the technology and systems used for telehealth for smooth running of the consultation and any troubleshooting^31,32,35,45,52–54^
Know how to collaborate effectively with service-users using relevant digital technologies^37,50^
Know how to test and troubleshoot your digital equipment and the service-users’ equipment^33,41,47^
Understand how your speech may be distorted through communication technologies^41^
Person-centered care
Be aware of how inadequate acceptance of telehealth by the service-user may negatively affect the validity and reliability of any remote assessments^47^
Ensure that the service-user provides informed consent on their digital data management, and any recording or sharing of any of their telehealth consultation^30,33,36,40,54^
Explicitly check that the service-user provides informed consent to receiving a telehealth consultation as acceptable^31,33,36^
Ensure that a therapeutic alliance is established and maintained throughout telehealth consultation^22^
Practice-based assessment and intervention knowledge, behaviour, and skills (KBSs)
Know how to choose the most appropriate technology for specific telehealth consultation contexts^24,31,32,36–39,45,50,55^
Privacy, security, and safety
Check that it is safe to conduct any telehealth assessments^33,36,42,51^
Provide explicit information on how the service-user’s digital data is managed, and how their privacy and confidentiality will be protected^23,22,33,39,41,53,54,56,57^
Provide information about any other persons that will be at any of the planned telehealth consultations^30,33,34,36,41^
Understand the terms and conditions of any software used in telehealth consultation to ensure data is kept confidential^54^
Professionalism
Have a professional environment and reduce clutter and other distractions from the background^22,21,36^
Keep updated on tax-related information for telehealth services^33^
Understand how to ensure the safety of service-users when engaging in telehealth^33,36^
Understand the limits of own competence when translating in-person service to telehealth^32,35,37,53^
Setting up the technical environment
Be aware of how sound and background noise can be measured accurately via telehealth compared to in-person^41^
Know how to efficiently set up technologies in preparation for any telehealth consultation^32^
Know how to regularly test your technology to ensure that it works appropriately and safely^23,38,42^

Table adapted from Anil et al.^9^ under the terms of the Creative Commons Attribution 4.0 License.^21^

**Table 4.** Competency Themes Relating to the Management of Telehealth Services

Telehealth service management
Digital infrastructure
Be aware of security and confidentiality risks associated with digital infrastructure^58^
Have a billing system that incorporates telehealth consultations and associated billing codes^34^
Have a central electronic health record with an understanding of the quality, impact, and use of data in practice^32,34,40^
Have an understanding of business systems and data related to telehealth service^32,33,44^
Understand the local digital infrastructure, policies, guidelines, and frameworks^23,33,40,41^
Informing practice
Keep up to date with relevant professional body best telehealth practices, research, and resources^31–33,35,36,41–43,47,48,51,52,56,57,59^
Know how to reflect on digital practices^33,37^
Know how to share and promote telehealth practice^20,37,60^
Understand how the differences in culture and social background can impact telehealth^19,23,24,21,30–32,34–38,47,53,57,60,61^
Understand how to implement best practices to ensure privacy and confidentiality for the service-user and others involved in the telehealth service^30–36,38–42,47,50,52,54,57,59,61,62^
Understand your local and national laws and scope of practice when engaging in telehealth^30–34,36,38,41,43,45,48,52,60^
Management
Ensure to have a mechanism in place where service-users are aware of their rights of accessing telehealth services including communication for complaints and grievances^42^
Ensure to maintain online professional boundaries^36,37,53^
Have a standard process for documentation^34^
Minimize barriers to accessibility of telehealth services for all service-user groups and providers^33,40^
Understand how to collaborate interprofessionally and with local providers to improve any telehealth service^19,25,28,18,26,32–34,40,41,44,50,51,57^
Understand how to plan and manage the delivery of telehealth services including contingencies and episodes of care^23,33,36,41,44,59^
Understand how to set up remote supervision of students or trainees^41^

Table reproduced from Anil et al.^9^ with no changes under the Terms of the Creative Commons Attribution 4.0 License.^21^

### Telehealth training and education

Table 5 presents details of the telehealth training and education reported within the included articles of this review, showing a variety of different training and education approaches. Some descriptions are more detailed than others, and some articles reported the teaching of one or two competencies while others taught several competencies. In addition, length of the training and education differed greatly across the included articles, and there was no definitive pattern in teaching approaches. Three articles^25,28,29^ used different standardized questionnaires to assess whether their participants acquired the targeted telehealth competencies.

**Table 5.** Training and Education Details Reported

Authors (date)	Cohort type (*n*)	Competencies targeted	Telehealth technologies targeted	Length of training	Training description	Interaction with service users	Training context description
Baalmann et al. (2023)^18^	Pharmacy and medical students (282)	Understand how to collaborate interprofessionally and with local providers to improve any telehealth service.	Online video software (Zoom)	Not specified	The training consisted of three phases: 1) Prelearning activities to prepare students for a telehealth simulation via a video of simulated service-users. 2) A telehealth consultation where a pharmacy student acted as a community pharmacist and a medical student as a hospital physician to address a case problem. 3) A debrief phase involving discussions and feedback sessions led by interprofessional faculty members.	Simulated service-users	Training provided during education and learning. Training assessed with author-designed survey.
Bautista et al. (2020)^19^	Pharmacy and medical students (5)	Understand how the differences in culture and social background can impact telehealth. Understand how to collaborate interprofessionally and with local providers to improve any telehealth service.	Telephone, online video software (Zoom)	Six sessions lasting 3 h across 2 weeks	The initial session was a 2-h orientation for students and faculty preceptors. It covered learning objectives, rotation activities, electronic medical record usage, and patient call procedures. Subsequent sessions followed a structured format: (1) preparing for simulation, (2) team collaboration via Zoom to review patient cases and address concerns, and (3) presenting telehealth experiences to faculty and scheduling follow-up appointments with administrative staff.	Real service-users	Training provided during education and learning. Training assessed with feedback survey and interview.
Bishop et al. (2013)^20^	Qualified physiotherapists (32)	Know how to assess during consultation whether face-to-face is required instead. Know how to effectively communicate via telephone as needed for a telehealth consultation. Know how to proactively and thoughtfully engage with the client using telehealth. Know how to share and promote telehealth practice.	Telephone	Two sessions lasting 2–3 h	The training occurred in three phases: 1. An intensive face-to-face session led by experienced PhysioDirect trainers. 2. Practice and skill consolidation using the computer-assisted system, with ongoing support from PhysioDirect trainers. 3. Competency checks conducted by PhysioDirect trainers, observing each physiotherapist assessing patients using the system.	None	Training provided during practice. No training assessment.
Crockett et al. (2020)^21^	Qualified Psychologists (117)	Ensure there is a plan to resolve the issue of suddenly becoming disconnected with the patient. Know how to address nonverbal cues when using telehealth. Have a professional environment and reduce clutter and other distractions from the background. Understand how the differences in culture and social background can impact telehealth.	Not reported	Not specified	All staff and trainees underwent self-paced, criterion-based competency training to reach a minimum competency level. Trainees were supervised during all telehealth appointments until minimum competencies were observed and documented. Then supervisors were available during all clinical appointments. Ongoing support was extended to other institute providers transitioning to telehealth. While the general preparations were consistent across disciplines, completing all required steps within a short time frame was challenging.	None	Training provided during practice. Training assessed with checklist.
Gafni-Lachter et al. (2023)^22^	Qualified mix of professionals working in pediatrics (120)	Know whether telehealth is an appropriate medium to communicate updates to service-users. Understand how to implement best practices to ensure privacy and confidentiality for the service-user and others involved in the telehealth service. Have a professional environment and reduce clutter and other distractions from the background. Know how to effectively communicate via telephone as needed for a telehealth consultation. Establish and maintain therapeutic alliance throughout telehealth consultation. Suitable selection and/or adaption of resources for telehealth.	Online education platform (not specified)	Ten weekly 3 h sessions	The course included weekly online lessons delivered. Each lesson included a live 2-h synchronous online lecture delivered by a content expert, followed by a 1 h asynchronous individual or group learning activities. These activities were shared in asynchronous online discussion boards to foster idea-sharing and networking and empower participants to translate actions into practice. Google Classroom was utilized for organization and delivery, while Zoom facilitated videoconferencing.	None	Training provided during education and learning. Training assessed with series of surveys.
Gifford et al. (2012)^23^	Qualified mix of professionals (21)	Know how to develop a contingency plan in case of technical failures and communicate this with the patient. Know how to address nonverbal cues when using telehealth. Provide explicit information on how the patient’s digital data are managed, and how their privacy and confidentiality will be protected. Know how to regularly test your technology to ensure that it works appropriately and safely. Understand the local digital infrastructure, policies, guidelines, and frameworks. Understand how the differences in culture and social background can impact telehealth. Understand how to plan and manage the delivery of telehealth services including contingencies and episodes of care.	Online video software (not specified)	Three full-day sessions	The training focused on identified set of core behavioral telehealth competencies that assumed a lack of familiarity with videoconferencing equipment and the nuances of providing behavioral health services via videoconferencing. Included practice with videoconferencing equipment, role playing clinical scenarios, and participation in small and large group discussions.	Not reported	Training provided during practice. Training assessed with pre-post mock interviews, self-rating of perceived competence, and follow-up survey.
McAllister et al. (2022)^24^	Speech and language therapy (SLT) students (34)	Understand how the differences in culture and social background can impact telehealth. Know how to choose the most appropriate technology for specific telehealth consultation contexts.	Online video software (Zoom), commercial online learning platform with videos and avatars.	6–7 weeks	This international course combined Vietnamese and non-Vietnamese students, with interpreters available throughout. The first 2-week training session involved intensive work with voice service-users in university SLT teaching clinics, with two telesupervisors. Students observed each other’s sessions and participated in planning and debriefing discussions. Prerecorded videos of telesupervisors working with service-users were available. The second placement period included online case-based learning due to service-user unavailability. International telesupervisors provided cases for discussion, covering service-user information, assessment priorities, tools, findings, and management strategies. Weekly Zoom meetings by the group leaders, for debriefing and planning.	Hybrid (simulated and real service-users)	Training provided during practice. Training assessed with student presentations and assessment of practice competency.
Pittman et al. (2023)^25^	Mixed student cohort (38)	Understand how to collaborate interprofessionally and with local providers to improve any telehealth service.	Online video software (not specified), telephone	Not specified	Not reported	Not reported	Training provided during placement. Training assessed with Team Skills Scale (TSS), a 17-item self-assessment measure of interprofessional team skills.
Rohrig et al. (2022)^26^	Mix of qualified professionals (800)	Understand how to collaborate interprofessionally and with local providers to improve any telehealth service	Online video software (not specified)	Not specified	Details unclear. Substantial online induction/preparation modules, plus 2.5–3 h per case (that includes orientation and debriefing).	Simulated service-users	Training provided during education and learning. Training assessed with performance assessment.
Rude et al. (2023)^27^	Pharmacy students (53)	Know how to effectively communicate via telephone as needed for a telehealth consultation	Telephone	1 h	1-h didactic lecture on professional telephone use and communication techniques and justifying changes to intervention.	Simulated service-users	Training provided during education and learning. Training assessed with pre-post survey.
Tokumaru et al. (2023)^28^	Pharmacy and nursing students (238)	Understand how to collaborate interprofessionally and with local providers to improve any telehealth.	Online video software (Zoom), PowerPoint, Google Docs, Telepresence Interprofessional Robot	2 full-day sessions	Nursing students interacted with a high-fidelity manikin, working with pharmacy students on another campus via a telepresence robot. They managed health care situations together, alternating between observing and actively participating in simulations. Faculty used detailed agendas, PowerPoint slides, and Google Docs to coordinate events and guide students during discharge planning meetings. Patient condition updates were presented using slides with images/static avatars and embedded audio clips. Facilitators simulated patient responses, transitioning the scenario into a telehealth encounter between nursing and pharmacy students.	Simulated service-users	Training provided during education and learning. Training assessed with interprofessional Collaboration Competency Attainment Survey, the modified National League for Nursing Simulation Design Scale, modified McMaster-Ottawa Individual Rating Scale.
Vyas et al. (2022)^29^	Pharmacy students (192)	Know how to proactively and thoughtfully engage with the client using telehealth.	Electronic health record, e-prescribing software, video recording software, and online video software (Zoom)	14 weeks	Seven weeks were synchronous with 2-h discussion sessions weekly. Course faculty used telehealth modalities for instruction delivery. Asynchronous sessions featured recorded lectures on topics such as service-user adherence, patient education, motivational interviewing, medication reconciliation, transitions of care, drug-related problems, and vaccine hesitancy; serving as preparation for telehealth simulations.	Simulated service-users	Training provided during education and learning. Training assessed with the Personal-Interpersonal Competence Assessment and a self-assessment rubric checked by supervisors.

### Outcomes

Two main outcomes were evident across the included articles. First, eight studies^18,19,22–25,28,29^ reported that their evaluated competencies were achieved through a training and education program. Second, five studies^19,22,23,25,27^ reported that participants’ attitudes toward telehealth, such as self-confidence and satisfaction when providing telehealth services, increased after training and education. These two outcomes highlight the potential benefit of a structured telehealth training and education program. However, not all studies reported that telehealth training and education was beneficial. Pittman et al.^25^ did not find a significant increase in teamwork-related competencies and suggest that this was because of a lack of in-person interaction during COVID-19 lockdown restrictions. This is in contrast to Baalman et al.’s^18^ finding where their participants, who were a mixed cohort of health professions students, scored the highest on team communication compared to other skills and knowledge. The reason for this contrast is unclear; however, a potential explanation may be due to the difference in training and methods used to assess their communication competency. In-person training,^28^ practice with real patients,^22^ and more intraprofessional discussions^22^ also appeared to benefit telehealth training and education.

### Gaps in research

According to authors of the included articles, the primary gap in the research was the evaluation of telehealth training and education implementation^19,21–23^ in authentic settings. Examining whether certain features would improve learning during training and education, such as the inclusion of video vignettes,^23^ scenarios with specific interventions,^28^ and simulations that allow for mistakes,^18^ were also suggested. Other gaps included understanding the nuances of telehealth training and education, such as assessing social and emotional development,^29^ and examining the impact on patient experience and telehealth service outcome.^25^

### Recommendations

Most recommendations from the included articles related to the features of telehealth training and education. One article recommended that all students should have the same level of telehealth understanding before engaging in any training and education.^28^ Telephone communication was suggested as needing its own distinct training element,^27^ and that a single video vignette should only include one or two telehealth competencies at most.^23^ Other recommendations stated that clinical experience is needed to deliver telehealth training and education,^20^ clinical staff should share telehealth service duties as staff do not want to solely work in telehealth,^20^ and that the evolution of technology should be monitored to ensure delivery of up-to-date telehealth services.^26^

### Article conclusions

Two studies emphasized the need to enhance telehealth training and education by incorporating a combination of online and face-to-face methods to optimize learning.^24,28^ Others concluded that interprofessional training facilitated communication learning^18,19,28^ through active teamwork, while students should have the opportunity to consolidate their skills through supervised clinical work,^20^ and telephone and video competencies should be separated.^20,27^ The remaining conclusions highlighted the need for organizational support^23^ and involvement of diverse stakeholder involvement^22^ for the successful development of telehealth training and education.

## Discussion

### Interpretation

This is the first scoping review to examine telehealth training and education across allied health disciplines and complements our previous review on allied health telehealth competencies^9^ where a paucity of literature in this field was identified. This review showcases the wide diversity of approaches to teaching telehealth competencies, with generally reported positive learning outcomes, despite no common teaching or evaluation method used across the included articles. There was limited rigorous evaluation of these programs and a lack of detail regarding the educational design and underpinning pedagogy. Consequently, it is unclear if the efficacy of the education relates to the quality of the instruction, the learning activity, or the degree of ease or difficulty in learning the targeted telehealth competencies. Furthermore, no pedagogical theories or frameworks were used in any of the articles to develop the telehealth training and education. It may, therefore, be possible that the education design and delivery influenced the learning outcomes. This prompts a call for an evidence-based curriculum design that can be adapted to the various allied health professions and education contexts. This would allow the acquisition of telehealth competencies to be the study focus and reduce the impact of the education design as a confounding variable.

An important consideration is the potential for bias within the included articles. The quality assessment showed that many studies did not sufficiently report on how bias was addressed within their study designs or outcomes. Specifically, the absence of established frameworks or theories, as noted above, may have introduced experience or profession-based biases. For example, the comfort or experience a participant may have with technology may have skewed results. AHPs often have distinct practices and priorities. Differences in professional outlooks, patient interactions, and disciplinary focus may lead to biases in how participants engage with and apply telehealth competencies. This highlights the need for future studies to adopt rigorous methodologies and reinforces the need for an evidence-based curriculum design.

### Comparison with previous studies

One study reported that in-person training^28^ was beneficial for teaching telehealth competencies, although the reason for this finding was unclear. It may be that a well-designed curriculum might not be affected by the delivery method, or there might be a methodological bias influencing this conclusion. Certain elements of telehealth training and education may be better suited for in-person teaching while others are well-suited for technology-enabled learning. This is reflected by the telehealth competency of determining suitability of telehealth versus in-person services, as demonstrated by Vyas et al.^29^ and Rohrig et al.^26^ as outlined in this review. Further research is needed to understand the features that optimize telehealth learning, and to determine when it is appropriate to use face-to-face teaching, technology, or a combination of both.

### Limitation

This scoping review has limitations. Although an extensive updated search was conducted, it targeted published, peer-reviewed literature. It is possible that there are more telehealth training and education articles that have not been published and hence not included. The search strategy was biased toward the English language, where non-English relevant articles may have been missed.

### Implications

The review findings indicate that telehealth competencies are an explicit skill set and need to be directly targeted by training and education. This is consistent with our previous scoping review findings that telehealth competencies need to be specifically taught.^9^ No articles in this review compared methods of education and training, making it difficult to conclude whether there is a “best” way of designing and implementing telehealth education and training. The competency themes identified here need further refinement before real-world implementation. By developing specific telehealth training alongside the refinement of these competency themes, explicit telehealth competencies and appropriate measures of success can be used to upskill AHPs in telehealth delivery.

## Conclusions

In conclusion, this scoping review represents the first comprehensive exploration of telehealth training and education across allied health disciplines. While diverse teaching approaches yielded generally positive outcomes, the absence of standardized methods prompts questions about its efficacy. Moreover, the emphasis of explicit telehealth competencies highlights the need for targeted training interventions. However, further refinement is necessary to ensure relevance in practice settings. Moving forward, research efforts should focus on developing evidence-based curriculum designs informed by high-quality pedagogic practices and tailored to AHP needs. By refining telehealth competencies and adopting appropriate measures of success, we can better prepare students and professionals for telehealth practice.

## Data Availability

None.

## Abbreviations Used

AHP: Allied Health Professions
KBS: Knowledge, behaviour, and skills
NHSE: National Health Service England
PCC: Population Concept Context
UK: United Kingdom
WHO: World Health Organisation
