# Supplementary material for: Telehealth Training and Education for Allied Health Professionals: A Scoping Review
Source: Telemed Rep. 2025 Mar 19;6(1):76–90. doi: 10.1089/tmr.2024.0083 (PMC12040532; doi:10.1089/tmr.2024.0083)
Supplement: Supplementary Data [file tmr.2024.0083_supplementary_data.docx]

**Supplementary Material – Updated Search Record**

**Record of Online Searches**

The below search strategy uses a logic grid and is based on the PCC framework. The searches were recorded and provided as an appendix with dates for any publications of the completed review.

| *Population* | *Concept 1* | *Concept 2* | *Context* |
| --- | --- | --- | --- |
| Art therap* | Tele* | Competenc* | Teach* |
| Audiolog* | Phone | Standard* | Learn* |
| Biomedical scien* | Video | Knowledge | Curriculum |
| Chiropract* | Remote | Skill* | Prac* |
| Chinese medic* | Simulat* | Behavio* | Course* |
| Clinical scien* |  |  | Prog* |
| Diabetes educat* |  |  | Module |
| Dietetics |  |  | Apprenticeship |
| Dietitian |  |  | Professional development |
| Diversional therap* |  |  |  |
| Drama therap* |  |  |  |
| Exercise scien* |  |  |  |
| Exercise physio* |  |  |  |
| Genetic counsel* |  |  |  |
| Hearing aid dispens* |  |  |  |
| Radiation oncology medical physics |  |  |  |
| Radiation therap* |  |  |  |
| Radiograph* |  |  |  |
| Music therap* |  |  |  |
| Occupational therap* |  |  |  |
| Operating department pract* |  |  |  |
| Optometr* |  |  |  |
| Orthopti* |  |  |  |
| Orthoti* |  |  |  |
| Prostheti* |  |  |  |
| Osteopath* |  |  |  |
| Paramedic* |  |  |  |
| Pedorthist |  |  |  |
| Perfusion* |  |  |  |
| Pharmac* |  |  |  |
| Physiotherap* |  |  |  |
| Podiatr* |  |  |  |
| Psycholog* |  |  |  |
| Rehabilitation counsel* |  |  |  |
| Social work* |  |  |  |
| Sonograph* |  |  |  |
| Speech therap* |  |  |  |
| Speech language therap* |  |  |  |

1. Each column was searched individually
2. All individual searches based on each column were combined with “AND”
3. Grey literature searches were tailored according to the individual database searching tools

*Database*: MEDLINE

*Date*: 28/07/2023

| **ID** | **Search Term(s)** | **Hits** |
| --- | --- | --- |
| 1 | Art therap* OR Audiolog* OR Biomedical scien* OR Chiropract* OR Chinese medic* OR Clinical scien* OR Diabetes educat* OR Dietetics OR Dietitian OR Diversional therap* OR Drama therap* OR Exercise scien* OR Exercise physio* OR Genetic counsel* OR Hearing aid dispens* OR Radiation oncology medical physics OR Radiation therap* OR Radiograph* OR Music therap* OR Occupational therap* OR Operating department pract* OR Optometr* OR Orthopti* OR Orthoti* OR Prostheti* OR Osteopath* OR Paramedic* OR Pedorthist OR Perfusion* OR Pharmac* OR Physiotherap* OR Podiatr* OR Psycholog* OR Rehabilitation counsel* OR Social work* OR Sonograph* OR Speech therap* OR Speech language therap* | 9,405,345 |
| 2 | Tele* OR Phone OR Video OR Remote OR Simulat* | 1,403,279 |
| 3 | Competenc* OR Standard* OR Knowledge OR Skill* OR Behavio* | 5,435,326 |
| 4 | Teach* OR Learn* OR Curriculum OR Prac* OR Course* OR Prog* OR Module OR Apprenticeship OR Professional development | 7,386,939 |
| 5 | ID1 AND ID2 AND ID3 AND ID4 | 5,222 |

Date limiter (July 2022) on ID 5

*Database*: CINHAL

*Date*: 28/07/2023

| **ID** | **Search Term(s)** | **Hits** |
| --- | --- | --- |
| 1 | Art therap* OR Audiolog* OR Biomedical scien* OR Chiropract* OR Chinese medic* OR Clinical scien* OR Diabetes educat* OR Dietetics OR Dietitian OR Diversional therap* OR Drama therap* OR Exercise scien* OR Exercise physio* OR Genetic counsel* OR Hearing aid dispens* OR Radiation oncology medical physics OR Radiation therap* OR Radiograph* OR Music therap* OR Occupational therap* OR Operating department pract* OR Optometr* OR Orthopti* OR Orthoti* OR Prostheti* OR Osteopath* OR Paramedic* OR Pedorthist OR Perfusion* OR Pharmac* OR Physiotherap* OR Podiatr* OR Psycholog* OR Rehabilitation counsel* OR Social work* OR Sonograph* OR Speech therap* OR Speech language therap* | 1,313,290 |
| 2 | Tele* OR Phone OR Video OR Remote OR Simulat* | 268,493 |
| 3 | Competenc* OR Standard* OR Knowledge OR Skill* OR Behavio* | 1,423,346 |
| 4 | Teach* OR Learn* OR Curriculum OR Prac* OR Course* OR Prog* OR Module OR Apprenticeship OR Professional development | 2,131,115 |
| 5 | ID1 AND ID2 AND ID3 AND ID4 | 902 |

Date limiter (July 2022) on ID 5

*Database*: EMBASE

*Date*: 28/07/2023

| **ID** | **Search Term(s)** | **Hits** |
| --- | --- | --- |
| 1 | Art therap* OR Audiolog* OR Biomedical scien* OR Chiropract* OR Chinese medic* OR Clinical scien* OR Diabetes educat* OR Dietetics OR Dietitian OR Diversional therap* OR Drama therap* OR Exercise scien* OR Exercise physio* OR Genetic counsel* OR Hearing aid dispens* OR Radiation oncology medical physics OR Radiation therap* OR Radiograph* OR Music therap* OR Occupational therap* OR Operating department pract* OR Optometr* OR Orthopti* OR Orthoti* OR Prostheti* OR Osteopath* OR Paramedic* OR Pedorthist OR Perfusion* OR Pharmac* OR Physiotherap* OR Podiatr* OR Psycholog* OR Rehabilitation counsel* OR Social work* OR Sonograph* OR Speech therap* OR Speech language therap* | 7,100,896 |
| 2 | Tele* OR Phone OR Video OR Remote OR Simulat* | 1,497,483 |
| 3 | Competenc* OR Standard* OR Knowledge OR Skill* OR Behavio* | 6,156,345 |
| 4 | Teach* OR Learn* OR Curriculum OR Prac* OR Course* OR Prog* OR Module OR Apprenticeship OR Professional development | 9,211,045 |
| 5 | ID1 AND ID2 AND ID3 AND ID4 | 5210 |

Date limiter (2022) on ID 5
